# A case of rapidly progressive Salmonella aortic aneurysm with acute pericarditis manifesting as a precursor

**DOI:** 10.1016/j.jccase.2024.11.006

**Published:** 2024-12-06

**Authors:** Koshiro Harada, Katsuya Kawagoe, Yunosuke Matsuura, Mana Kawano, Yosuke Suiko, Hiroki Tanaka, Kohei Moribayashi, Hirohito Ishii, Takeshi Ideguchi, Koji Furukawa, Koichi Kaikita

**Affiliations:** a Division of Cardiovascular Medicine and Nephrology, Department of Internal Medicine, Faculty of Medicine, University of Miyazaki, Miyazaki, Japan; b Department of Cardiovascular Surgery, Faculty of Medicine, University of Miyazaki, Miyazaki, Japan

**Keywords:** Acute pericarditis, Infectious aortic aneurysm, Salmonella

## Abstract

We report a case of Salmonella cardiovascular infection presenting with acute pericarditis as a precursor to the rapid progression of aortic aneurysm. An 81-year-old man presented with persistent fever and chest pain worsened with inspiration and was admitted to a nearby hospital with a diagnosis of bacterial pericarditis. However, hoarseness emerged two days later, and the patient was transferred to our hospital because of concerns about extracardiac inflammatory foci. Computed tomography (CT) revealed a periaortic exudate and aortic arch aneurysm. After transfer, blood cultures confirmed Salmonella infection. Ampicillin (ABPC) was initiated for long-term treatment of Salmonella infection, and pericarditis was treated with ibuprofen and colchicine for approximately one month. The associated symptoms and inflammatory blood data significantly improved, but five weeks later, follow-up CT revealed enlargement of the arch aneurysm. Due to the patient's age and nutritional status, thoracic endovascular aortic repair (TEVAR) was performed along with continued ABPC. Postoperatively, the infection was well-controlled, and follow-up CT revealed a size reduction in the treated aneurysm. No recurrent Salmonella-related vascular events were observed for two years after TEVAR.

**Learning objective:**

Acute pericarditis can present as a precursor to life-threatening vascular lesions associated with Salmonella infection and requires timely and appropriate diagnosis of the etiology behind the manifestation. Patients with aortic aneurysms caused by Salmonella often do not tolerate invasive surgical treatment when diagnosed, and the lesions progress rapidly. Therefore, endovascular treatment combined with long-term antibiotic therapy may be a practical option.

## Introduction

Acute pericarditis is generally a self-limiting pericardially localized disease [1], but it can occasionally manifest as part of a progressive inflammatory process involving the cardiovascular system. In such cases, timely diagnosis of the underlying etiology of acute pericarditis and subsequent appropriate treatment may be necessary to reduce the risk of acute complications [2].

Salmonella-associated cardiovascular diseases also include pericarditis and aortic aneurysms, which are often challenging to diagnose due to their diverse clinical presentations. Therefore, a more comprehensive understanding of their clinical scenarios is essential for improving clinical outcomes.

In this report, we present a case of Salmonella cardiovascular infection presenting with acute pericarditis as a precursor to the rapid progression of aortic aneurysm.

## Case report

**Fig. 1:**
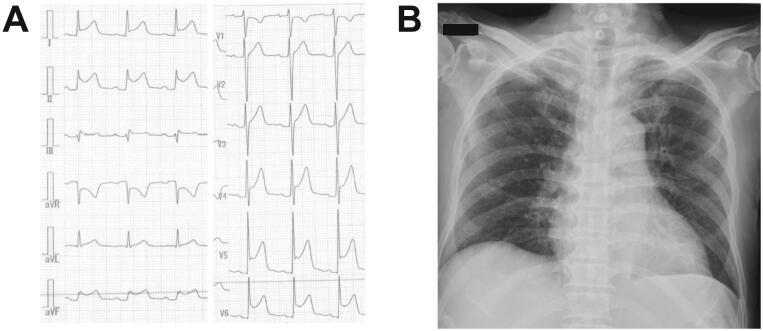
Electrocardiogram (A) and chest-X ray (B) on admission.

An 81-year-old man with hypertension and paroxysmal atrial fibrillation was admitted to our hospital with persistent fever and anterior chest pain that had worsened on inspiration for one month. Electrocardiography showed extensive ST-segment elevation in the precordial and inferior leads (Fig. 1A) and white blood cell counts, C-reactive protein, and procalcitonin levels were elevated. Chest X-ray showed a mild increase in the cardiothoracic ratio but no apparent pulmonary lesions or mediastinal enlargement (Fig. 1B). Echocardiography did not reveal valvular abnormalities suggestive of infective endocarditis, but limited pericardial effusion was observed. Before administering antibiotics, blood culture tests were performed, and ceftriaxone was empirically initiated as a diagnosis of bacterial pericarditis. However, two days later, hoarseness suddenly appeared without any apparent trigger, and the patient was transferred to our hospital for further examination because of concerns regarding the presence of extracardiac inflammatory foci.

Upon admission to our hospital, the patient's temperature was 37.7 °C, heart rate was 91 beats/min, blood pressure (BP) was 130/84 mmHg, and SpO2 was 97 %. We found a slight pericardial friction sound but no jugular vein dilatation or peripheral edema. Contrast-enhanced computed tomography (CT) revealed an exudate effusion around the aortic arch to the descending aorta and a 10-mm aneurysm with irregular protrusion towards the cephalic side of the aortic wall. Additionally, consistent with the echocardiographic findings at our hospital (Video 1), the pericardium was mildly thickened, and a small amount of pericardial effusion was observed. After the transfer, a previous physician reported that the blood culture was positive for Salmonella. A detailed interview revealed that the patient had a history of fever and frequent diarrhea for three weeks after ingesting chicken eggs two months before admission, but the symptoms resolved spontaneously.

Ampicillin (ABPC) was initiated with the diagnosis of Salmonella infection, and ibuprofen and colchicine were co-administered to treat acute pericarditis. After the above treatment, fever, chest pain, and hoarseness disappeared immediately, and the inflammatory data improved. The patient's blood pressure was well controlled, and follow-up CT was regularly performed to check for aneurysm enlargement.

**Fig. 2:**
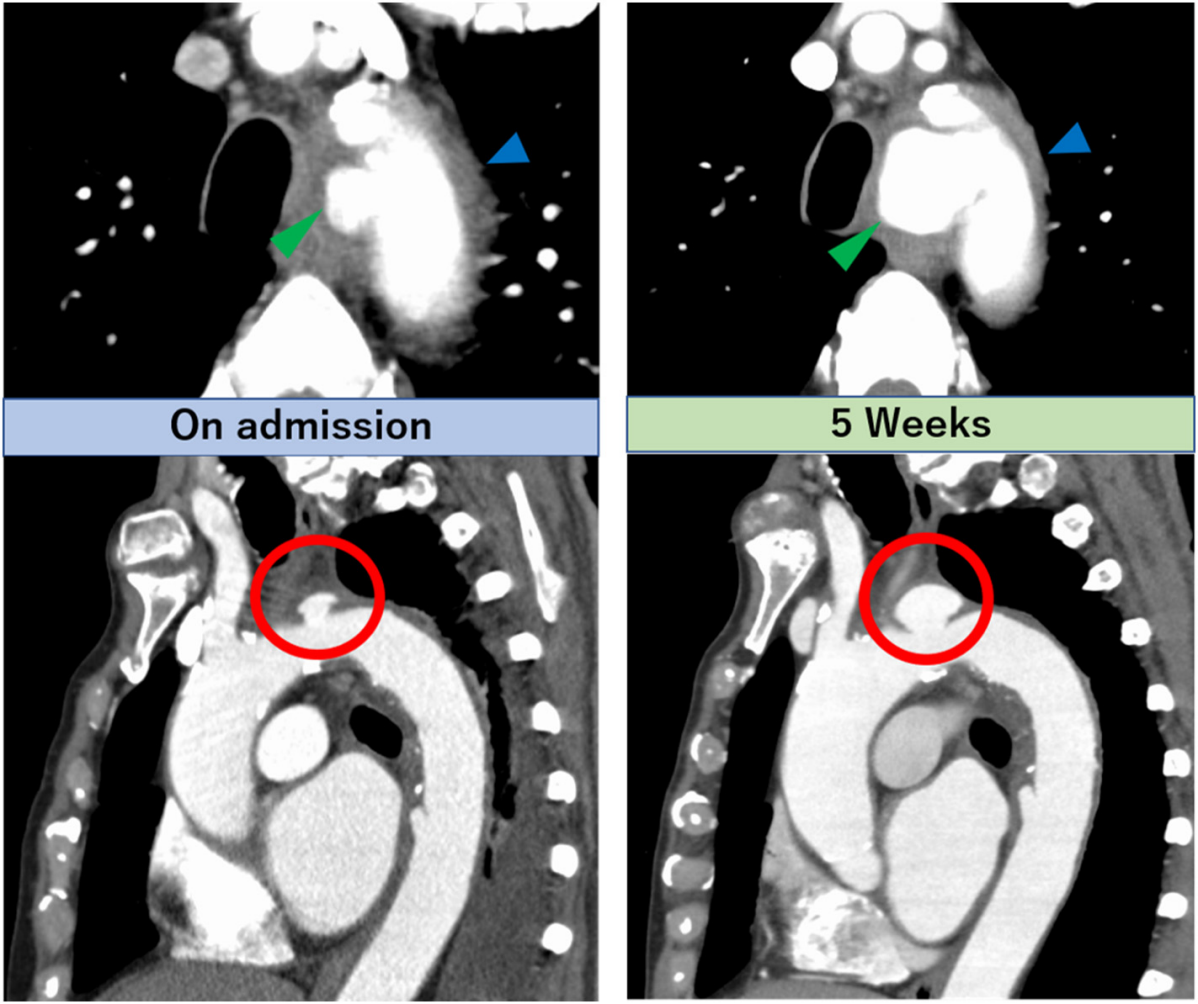
Contrast-enhanced CT on admission and after five weeks of antibiotics. *Green arrow*: aortic arch aneurysm (cross-sectional image); *blue arrow*: periaortic arch effusion; *red circle*: aortic arch aneurysm (sagittal image). CT, computed tomography.

**Fig. 3:**
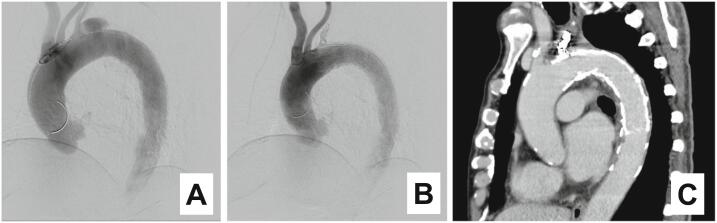
(A) Pre-TEVAR Aortography; (B) post-TEVAR Aortography; (C) post-TEVAR CT. TEVAR, thoracic endovascular aortic repair; CT, computed tomography.

Five weeks after admission, CT showed that the small, localized aneurysm had approximately doubled in size (diameter, 21 mm) (Fig. 2). However, due to older age and poor nutritional status, the patient was at high surgical risk, and thoracic endovascular aortic repair (TEVAR) along with left subclavian artery coil embolization was planned and successfully performed under continued antibiotic therapy (Fig. 3A-C). Specifically, for the size of the stent-grafts, a 37-mm stent graft for a proximal vessel diameter of 31 mm and a 30-mm stent graft for a distal vessel diameter of 28 mm was selected, respectively. The length of the stent graft was determined based on the requirements to cover the aneurysm in the aortic arch, and the ulcer-like projection in the descending aorta. Axillary-axillary bypass was avoided in the acute phase because of concerns about the risk of graft vessel infection. The bilateral vertebral arteries, including the basilar artery, were well perfused on preoperative evaluation, and the left subclavian artery showed satisfactory perfusion on final postoperative angiography, which provided the background that enables the avoidance of axillary-axillary bypass. In addition, intraoperative and postoperative BP was managed with the goal of a mean BP >80 mmHg to maintain perfusion pressure in vessels located in the spinal cord, and the absence of paralytic symptoms was confirmed as soon as possible after TEVAR. If signs of paralysis appeared, the patient had been prepared to undergo an urgent axillary-axillary bypass; however, a stable clinical course was observed without any events. The infection status and inflammatory laboratory data were also well-controlled postoperatively. Additionally, the follow-up CT revealed a size reduction in the treated aneurysm. Due to left upper extremity fatigue from subclavian artery coil embolization, axillo-axillary bypass was required in the chronic phase; however, no recurrence of clinical vascular events related to Salmonella infection was observed for two years after TEVAR.

## Discussion

We report a successfully treated case of a rapidly progressing Salmonella aortic aneurysm, with acute pericarditis manifesting as a precursor. Infectious aortic aneurysms are rare; however, without proper management based on accurate diagnosis, the disease progresses rapidly and poses a severe threat to life [3]. Although Salmonella is a common pathogen associated with infectious aortic aneurysms [4], diagnosing cardiovascular infections caused by Salmonella is often challenging because of the diverse and dynamic nature of its pathogenesis [5]. The patient also had acute pericarditis as the initial manifestation, followed by hoarseness, and a CT tomography revealed vascular lesions. However, whether vascular or pericardial inflammatory lesions first develop in the subclinical stage is unclear. Salmonella-related vascular infections are generally associated with preexisting atherosclerotic lesions [6], and Salmonella is postulated to have invasive infiltration properties into compromised vessel walls [7]. Therefore, careful follow-up of vascular lesions may be mandatory for Salmonella infections, even if temporarily limited to symptoms of pericarditis.

However, even when diagnosed with Salmonella aortic aneurysms, patients are often elderly and malnourished [8], and invasive interventions are frequently impractical. In our case, rapid aneurysm enlargement was observed during CT follow-up, resulting in the decision that some type of vascular intervention was necessary. Consequently, TEVAR was selected as the less invasive approach to prevent rupture. According to the guidelines, endovascular treatment of infected aneurysms was considered a contraindication to surgery but is now indicated as a form of bridging therapy [9]. Previous evidence has shown that endovascular repair with antibiotics can provide a favorable long-term clinical course in infectious aortic aneurysms [10], similar to our case. Long-term antibiotic therapy was administered in our case; however, no consensus exists as to how long it should be administered. Further studies are required to determine the optimal duration of antibiotic therapy.

In summary, acute pericarditis followed by hoarseness led to the suspicion of extracardiac inflammatory foci. CT imaging enabled the early diagnosis of Salmonella aortic aneurysm, effectively preventing aneurysm rupture by TEVAR in the patient. Thus, Salmonella infection with vascular lesions can be a differential diagnosis for the underlying etiology of acute pericarditis.

The following is the supplementary data related to this article.

Video 1 Echocardiography on admission. Video 1

## Consent statement

Written informed consent was obtained from the patient to publish this case report.

## Funding

None.

## Declaration of competing interest

The authors declare that there is no conflict of interest.
